# Supplementary material for: A prion accelerates proliferation at the expense of lifespan
Source: eLife. 2021 Sep 21;10:e60917. doi: 10.7554/eLife.60917 (PMC8455135; doi:10.7554/eLife.60917)
Supplement: Supplementary file 1. [file elife-60917-supp1.docx]

**Supplementary file 1:** Parameter values used for the competitive fitness models shown in **Figure 2** and **Figure 2—figure supplement 1.**

| **Parameter** | **Meaning** | **Value** |
| --- | --- | --- |
| *x_0_* | number of *LS-DO* cells | n/a |
| *x_1_* | number of *LF-DY* cells | n/a |
| *x_00_* | Initial number of *LS-DO* cells | 1 |
| *x_10_* | Initial number of *LS-DO* cells | 1 |
| *μ_0_* | Growth rate of *LS-DO* cells | 1 |
| *μ_1_* | Growth rate of *LF-DY* cells | 1.01 |
| *δ_0_* | Death rate of *LS-DO* cells | 1 |
| *δ_1_* | Death rate of *LS-DO* cells | 1.01 |
| *τ_1_* | Time of nutrient repletion | 1 |
| *τ_2_* | Time of starvation | 1 |
