# Supplementary material for: A prion accelerates proliferation at the expense of lifespan
Source: eLife. 2021 Sep 21;10:e60917. doi: 10.7554/eLife.60917 (PMC8455135; doi:10.7554/eLife.60917)
Supplement: Transparent reporting form [file elife-60917-transrepform1.docx]

***eLife’s* transparent reporting form**

We encourage authors to provide detailed information *within their submission* to facilitate the interpretation and replication of experiments. Authors can upload supporting documentation to indicate the use of appropriate reporting guidelines for health-related research (see [EQUATOR Network](http://www.equator-network.org/%20)), life science research (see the [BioSharing Information Resource](https://biosharing.org/)), or the [ARRIVE guidelines](http://www.plosbiology.org/article/info:doi/10.1371/journal.pbio.1000412) for reporting work involving animal research. Where applicable, authors should refer to any relevant reporting standards documents in this form.

If you have any questions, please consult our Journal Policies and/or contact us: [editorial@elifesciences.org](mailto:editorial@elifesciences.org).

**Sample-size estimation**

- You should state whether an appropriate sample size was computed when the study was being designed
- You should state the statistical method of sample size computation and any required assumptions
- If no explicit power analysis was used, you should describe how you decided what sample (replicate) size (number) to use

Please outline where this information can be found within the submission (e.g., sections or figure legends), or explain why this information doesn’t apply to your submission:

Sample sizes are listed in figure legends, Materials and Methods, and sometimes within the main text. Every experiment contains three or more biological replicates (definition below), and often within each biological replicate, from dozens (e.g. replicative lifespan measurements) to thousands (e.g. microscopy to measure cell area/size) of cells were analyzed.

**Replicates**

- You should report how often each experiment was performed
- You should include a definition of biological versus technical replication
- The data obtained should be provided and sufficient information should be provided to indicate the number of independent biological and/or technical replicates
- If you encountered any outliers, you should describe how these were handled
- Criteria for exclusion/inclusion of data should be clearly stated
- High-throughput sequence data should be uploaded before submission, with a private link for reviewers provided (these are available from both GEO and ArrayExpress)

Please outline where this information can be found within the submission (e.g., sections or figure legends), or explain why this information doesn’t apply to your submission:

Number of replicates are listed in figure legends, Materials and Methods, and sometimes within the main text. Biological replicates are measurements deriving from different yeast colonies, while technical replicates are independent measurements deriving from biological material that can be traced back to a single yeast colony. The GEO link for RNA-sequencing data is given in Materials and Methods.

**Statistical reporting**

- Statistical analysis methods should be described and justified
- Raw data should be presented in figures whenever informative to do so (typically when N per group is less than 10)
- For each experiment, you should identify the statistical tests used, exact values of N, definitions of center, methods of multiple test correction, and dispersion and precision measures (e.g., mean, median, SD, SEM, confidence intervals; and, for the major substantive results, a measure of effect size (e.g., Pearson's r, Cohen's d)
- Report exact p-values wherever possible alongside the summary statistics and 95% confidence intervals. These should be reported for all key questions and not only when the p-value is less than 0.05.

Please outline where this information can be found within the submission (e.g., sections or figure legends), or explain why this information doesn’t apply to your submission:

Statistical analysis methods and p values are listed in figure legends, Materials and Methods, and sometimes within the main text. When multiple measurements are conducted from multiple biological measurements, a data point representing the mean of all measurements within a biological replicate are displayed, for clarity. The mean of means is then displayed as, for example, the bar height. An additional “statistical reporting” .xlsx document, includes, for each figure panel and when applicable, the error represented, the statistical test, the sample comparison used to compute the test, and the p value.

(For large datasets, or papers with a very large number of statistical tests, you may upload a single table file with tests, Ns, etc., with reference to sections in the manuscript.)

**Group allocation**

- Indicate how samples were allocated into experimental groups (in the case of clinical studies, please specify allocation to treatment method); if randomization was used, please also state if restricted randomization was applied
- Indicate if masking was used during group allocation, data collection and/or data analysis

Please outline where this information can be found within the submission (e.g., sections or figure legends), or explain why this information doesn’t apply to your submission:

Not applicable to this study.

**Additional data files (“source data”)**

- We encourage you to upload relevant additional data files, such as numerical data that are represented as a graph in a figure, or as a summary table
- Where provided, these should be in the most useful format, and they can be uploaded as “Source data” files linked to a main figure or table
- Include model definition files including the full list of parameters used
- Include code used for data analysis (e.g., R, MatLab)
- Avoid stating that data files are “available upon request”

Please indicate the figures or tables for which source data files have been provided:

Supplementary file 1 – Model definitions and parameters (MatLab code and data required to generate the model predictions is available at: github.com/cjakobson/liveFastDieYoung)

Supplementary file 2 – RNA-sequencing data

Supplementary file 3 – GFP screen hits

Supplementary file 5 – Pus4 targets, position weight matrix
